# Comparative efficacy of oral insulin sensitizers metformin, thiazolidinediones, inositol, and berberine in improving endocrine and metabolic profiles in women with PCOS: a network meta-analysis

**DOI:** 10.1186/s12978-021-01207-7

**Published:** 2021-08-18

**Authors:** Han Zhao, Chuan Xing, Jiaqi Zhang, Bing He

**Affiliations:** Department of Endocrinology, Shengjing Hospital, China Medical University, Shenyang, 110000 Liaoning People’s Republic of China

**Keywords:** Polycystic ovary syndrome, Oral insulin sensitizers, Metformin, Thiazolidinediones, Inositol, Berberine, Endocrine, Metabolic, Network meta-analysis

## Abstract

**Background:**

Multiple oral insulin-sensitizing agents, such as metformin, thiazolidinediones, inositols, and berberine, have been proven safe and efficacious in improving the endocrine, metabolic, and reproductive abnormalities seen in polycystic ovary syndrome (PCOS), providing more options for healthcare providers and patients. These oral insulin sensitizers are more convenient, practical, and economic than agents that need to be injected. A comparison of the clinical effectiveness of the four different classes of oral insulin sensitizers in PCOS has not been explored, leading to clinical uncertainty about the optimal treatment pathway. The present study aims to compare the effects of oral insulin sensitizers on endocrine and metabolic profiles in women with PCOS.

**Methods:**

We identified randomized controlled trials for PCOS from a variety of databases, published from January 2005 to October 2020. Outcomes included changes in menstrual frequency, improvements in hyperandrogenism and glucolipid metabolism and adverse side effects. A random-effects network meta-analysis was performed.

**Results:**

Twenty-two trials comprising 1079 patients with PCOS were included in this study. Compared with metformin, treatment with myo-inositol + d-chiro-inositol was associated with a greater improvement in menstrual frequency (odds ratio 14.70 [95% confidence interval (CI) 2.31–93.58]). Myo-inositol + d-chiro-inositol and metformin + thiazolidinediones combination therapies were superior to respective monotherapies in reducing total testosterone levels. Thiazolidinediones, metformin + thiazolidinediones, and myo-inositol + d-chiro-inositol were associated with a lower insulin resistance index (HOMA-IR) compared with that in metformin alone (mean differences: − 0.72 [95% CI (− 1.11)–(− 0.34)] to − 0.89 [95% CI (− 1.460)–(− 0.32)]). Metformin + thiazolidinediones treatment was associated with lower triglyceride levels compared with that in metformin and thiazolidinediones monotherapy, while thiazolidinediones was superior to metformin in increasing high-density lipoprotein cholesterol and decreasing fasting plasma glucose, triglycerides, low-density lipoprotein cholesterol, and gastrointestinal adverse events.

**Conclusions:**

Ours is the first study to report that for women with PCOS, myo-inositol combined with d-chiro-inositol and metformin combined with thiazolidinediones appear superior to metformin alone in improving insulin resistance and decreasing total testosterone. Myo-inositol combined with d-chiro-inositol is particularly efficacious in menstrual recovery. Thiazolidinediones and metformin combined with thiazolidinediones improve lipid metabolism better than metformin alone.

*Trial registration* PROSPERO CRD42020211524

**Supplementary Information:**

The online version contains supplementary material available at 10.1186/s12978-021-01207-7.

## Plain English summary

This study aimed to compare the effects of oral insulin sensitizers on endocrine and metabolic profiles in women with polycystic ovary syndrome (PCOS). A random-effects network meta-analysis including 22 trials was conducted. For women with PCOS, myo-inositol combined with d-chiro-inositol and metformin combined with thiazolidinediones appear superior to metformin alone in improving insulin resistance and decreasing total testosterone level. Myo-inositol combined with d-chiro-inositol is particularly efficacious in menstrual recovery. Thiazolidinediones and metformin combined with thiazolidinediones improve lipid metabolism better than metformin alone.

**Supplementary Information:**

The online version contains supplementary material available at 10.1186/s12978-021-01207-7.

## Background

Polycystic ovary syndrome (PCOS) is a common and complex endocrinopathy that affects 4–21% of women of reproductive age worldwide [1, 2]. It is characterized by ovulatory dysfunction, hyperandrogenism, and a polycystic ovarian morphology. These features are accompanied by various metabolic abnormalities, such as insulin resistance, hyperinsulinemia, and adiposity. PCOS has a long-term impact on overall health, such as an increased risk of endometrial cancer, type 2 diabetes mellitus, and cardiovascular events [3, 4]. Metformin (Met), a recognized insulin sensitizer, has been widely used for women with PCOS due to benefits, such as improving menstruation and hyperinsulinemia, hyperandrogenism, and abnormal metabolism, and it may also have a preventive effect on long-term cardiovascular diseases [5]. However, the persistent use of Met is accompanied by gastrointestinal adverse side effects, such as diarrhea and stomachache [6]. Multiple oral insulin-sensitizing agents, such as thiazolidinediones (TZDs), inositols, and berberine, have been proven safe and efficacious in improving the endocrine, metabolic, and reproductive abnormalities seen in PCOS, providing more options for healthcare providers and patients. These oral insulin sensitizers are more convenient, practical, and economic than agents that need to be injected. However, a comparison of the clinical effectiveness of the four different classes of oral insulin sensitizers in PCOS has not been explored, leading to clinical uncertainty about the optimal treatment pathway. Here, a network meta-analysis (NMA) was designed using metformin as control to compare the efficacy of the four different classes of oral insulin sensitizers in improving menstruation, hyperandrogenism, and abnormal metabolism in patients with PCOS, along with an assessment of their relative safety profiles.

## Materials and methods

The methods and results are reported following the Preferred Reporting Items for Systematic Reviews and Meta-Analyses statement [7]. The protocol was registered at PROSPERO as CRD42020211524.

### Search strategy and selection criteria

Systematic literature searches were performed in the following databases from January 2005 to October 23rd, 2020: PubMed, Embase, Web of Science, the Cochrane Central Register of Controlled Trial (CENTRAL), CBM Database, CNKI Database, the WanFang Database, the WeiPu Database, and ClinicalTrials.gov. We used different combinations of the following search terms: “polycystic ovary syndrome,” “metformin,” “thiazolidinediones,” “inositol,” and “berberine.” The PICO framework was used to identify relevant trials [8], without language, ethnicity, or regional restrictions. We included randomized controlled trials (RCTs) in women, aged 18 to 49 years, diagnosed with PCOS based on the Rotterdam consensus, the Androgen Excess and PCOS Society criteria, the National Institute of Health, and Guidelines for diagnosis and treatment of PCOS in China [9–12]. Eligible trials included those where treatments were followed-up for at least 12 weeks, consisting at least one of the predefined outcomes namely, menstruation, hyperandrogenism, and metabolism, and comparison of the effects of insulin sensitizers. Additionally, treatments combined with either oral contraceptives or ovulation-inducing agents, and patients with other diseases, such as nonclassic congenital adrenal hyperplasia, premature ovarian failure, hyperprolactinemia, hypothyroidism, hyperthyroidism, Cushing’s syndrome, and androgen-secreting tumors were excluded.

### Outcomes

The outcomes included menstruation frequency, parameters of hyperandrogenism [total testosterone (TT), sex hormone-binding globulin (SHBG), androstenedione (AND), and the modified Ferriman–Gallwey score (mF-G score)], parameters of glucolipid metabolism, including fasting plasma glucose (FPG), fasting insulin (FINS), Homeostatic Model Assessment of Insulin Resistance (HOMA-IR), and lipids [triglyceride (TG), total cholesterol (TC), high-density lipoprotein cholesterol (HDL-C), and low-density lipoprotein cholesterol (LDL-C)]. Obesity-related indexes, such as body mass index (BMI) and waist–hip ratio (WHR), and adverse events were also considered.

### Data extraction and quality assessment

Two independent reviewers (H.Z. and C.X.) screened the titles and abstracts and assessed the full texts of potential reports. The data extracted from the original trials included study, year, region, intervention, sample size, follow-up duration, and outcomes of interest (Table 1). Risk of bias assessment was assigned by two independent investigators (H.Z. and J.Z.) using the revised Cochrane Collaboration risk of bias tool RoB 2.0 across five domains [35]. Each trial was evaluated by two reviewers (H.Z. and C.X.), any discrepancies were resolved by a consensus-based discussion with another author (B.H.).

**Table 1:** Characteristics of studies included in the network meta-analysis

Study	Year	Region	Drugs				Size	F-up (wks)	Efficacy
Ahmad [13]	2008	India	Met vs TZDs	Met	850 mg	bid	31	24 w	FPG, FINS, HOMA-IR, BMI, WHR, TT, AND, mF-G score, Menstrual frequency, Adverse events
				Rosi	2 mg	bid	30		
Mohiyididden [14]	2013	UK		Met	500 mg	bid	17	12 w	FPG, FINS, TC, TG, HDL, LDL, BMI, TT, SHBG, Menstrual frequency, Adverse events
				Rosi	4 mg	qd	18		
Yilmaz [15]	2005	Turkey		Met	850 mg	bid	25	12 w	BMI, WHR, AND, HOMA-IR, TC, TG, LDL, HDL, Menstrual frequency, Adverse events
				Rosi	4 mg	qd	25		
Sangeeta [16]	2012	India		Met	500 mg	bid	50	24 w	FINS, HOMA-IR, TC, HDL, mF-G score, TT, SHBG, Menstrual frequency, Adverse events
				Pio	15 mg	qd	50		
Naka [17]	2011	Greece		Met	850 mg	bid	15	24 w	FPG, FINS, TC, TG, HDL, LDL, mF-G score, TT, SHBG, BMI, WHR, Adverse events
				Pio	30 mg	qd	14		
Jensterl [18]	2008	Slovenia		Met	850 mg	bid	15	24 w	FPG, FINS, HOMA-IR, TC, TG, HDL, LDL, BMI, TT, AND, Menstrual frequency, Adverse events
				Rosi	4 mg	qd	11		
Ortega [19]	2005	Mexico		Met	850 mg	tid	18	24 w	FPG, FINS, HOMA-IR, TC, TG, HDL, LDL, BMI, WHR, mF-G score, AND, Adverse events
				Pio	30 mg	qd	17		
Zeng [20]	2020	China	Met vs Met + TZDs	Met	500 mg	tid	44	12 w	FPG, FINS, HOMA-IR, TC, TG, HDL, LDL, BMI, TT, Adverse events
				Met + Pio	500 mg + 15 mg	bid	44		
Wang X [21]	2014	China		Met	1000 mg	tid	43	24 w	FPG, FINS, HOMA-IR, HDL, LDL, BMI, WHR, TT, SHBG, mF-G score, Menstrual frequency, Adverse events
				Met + Pio	1000 mg + 5 mg	tid	43		
Liang [22]	2019	China	Met vs TZDs vs Met + TZDs	Met	500 mg	tid	22	12 w	FPG, FINS, HOMA-IR, HDL, LDL, TC, TG, BMI, WHR, TT, Menstrual frequency
				Pio	30 mg	tid	21		
				Met + Pio	500 mg + 30 mg	tid	23		
Sohrevardi [23]	2016	Iran		Met	500 mg	tid	22	12 w	PFG, FINS, HOMA-IR, TC, TG, HDL, LDL, BMI, TT, WHR, Menstrual frequency, Adverse events
				Pio	30 mg	qd	21		
				Met + Pio	500 mg + 30 mg	tid/qd	23		
Jamilian [24]	2017	Iran	Met vs MI	Met	500 mg	tid	30	12 w	BMI, SHBG, TT, mF-G score
				MI	2 g	bid	30		
Shokrpour [25]	2019	Iran		Met	500 mg	tid	27	12 w	FPG, FINS, HOMA-IR, BMI, TC, TG, HDL-C, LDL-C
				MI	2 g	bid	26		
Fruzzetti [26]	2016	Italy		Met	500 mg	tid	22	24 w	HOMA-IR, BMI, mF-G score, Menstrual frequency, Adverse events
				MI	4 g	qd	24		
Nehra [27]	2017	India		Met	500 mg	tid	30	12–24 w	FPG, FINS, HOMA-IR, TC, TG, HDL, LDL, TT, BMI, WHR
				MI	1 g	bid	30		
Du [28]	2018	China	Met vs MI + DCI	Met	500 mg	bid	32	24 w	FPG, FINS, HOMA-IR, TC, TG, HDL, LDL, AND, TT, SHBG, Menstrual frequency, Adverse events
				MI + DCI	550 mg + 13.8 mg	bid	32		
Pizzo [29]	2014	Italy	MI vs DCI	MI	4 g	qd	25	24 w	HOMA-IR, BMI, mF-G score, TT, AND, SHGB, Menstrual frequency
				DCI	1 g	qd	25		
Donne [30]	2019	Italy	MI vs MI + DCI	MI	4 g	qd	10	12–24 w	mF-G score, WHR, BMI, Menstrual frequency
				MI + DCI	1.1 g + 27.6 mg	qd	12		
Nordio [31]	2012	Italy		MI	2 g	bid	24	12–24 w	BMI, WHR, FPG, FINS, HOMA-IR, TT, SHBG, AND, Menstrual frequency
				MI + DCI	550 mg + 13.8 mg	bid	26		
Li [32]	2017	China	Met vs BBR	Met	500 mg	bid	29	12 w	FPG, FINS, HOMA-IR, TC, TG, HDL, LDL, BMI, TT, Adverse events
				BBR	300 mg	tid	26		
Wang P [33]	2016	China	Met vs Met + BBR	Met	500 mg	tid	42	12 w	HOMA-IR, BMI, WHR
				Met + BBR	500 mg + 500 mg	tid	42		
Wang L [34]	2011	China		Met	500 mg	tid	28	12 w	FPG, FINS, HOMA-IR, BMI, TT
				Met + BBR	500 mg + 500 mg	tid	28		

*Met* Metformin, *TZDs* thiazolidinediones, *Rosi* rosiglitazone, *Pio* pioglitazone, *MI* myo-inositol, *DCI* d-chiro-inositol, *BBR* berberine, *TT* total testosterone, *SHBG* sex hormone binding globulin, *AND* androstenedione, *mF-G score* modified Ferriman–Gallwey score, *BMI* body mass index, *WHR* waist–hip ratio, *FPG* fasting plasma glucose, *FINS* fasting insulin, *HOMA-IR* Homeostatic Model Assessment of Insulin Resistance, *TG* triglyceride, *TC* total cholesterol, *HDL-C* high density lipoprotein cholesterol, *LDL-C* low density lipoprotein cholesterol

### Data synthesis and analyses

The efficacy of different treatment regimens was compared simultaneously using a traditional pairwise meta-analysis (TMA) and network meta-analysis (NMA) [36]. Initially, we conducted a random-effect TMA using Review Manager 5.4 (the Cochrane Collaboration, London, UK). Continuous variables were represented by the weighted mean differences (MDs) with 95% confidence intervals (CIs), and for dichotomous variables, we calculated the combined odds ratios (ORs) with 95% CIs [37]. Between-study heterogeneity was determined using Chi-squared test, combining *I*^2^ and *P* values, for which *I*^2^ > 50% or *P* < 0.05, indicated substantial heterogeneity. The random-effect NMA was subsequently performed to combine the direct and indirect comparisons of agents into one analysis with Stata software (version 15.1, Stata Corp LLC, 4905 Lakeway Drive, College Station, TX, USA). We calculated the pooled estimates of MDs or ORs with 95% CIs in order to compare multiple interventions to each other. Network inconsistency was evaluated using the node-splitting method and inconsistency models, and significance was assessed at the 0.05 level. Finally, to summarize the probabilities, we calculated the surface under the cumulative ranking (SUCRA) curve to provide a summary statistic for cumulative ranking. The efficacy of each intervention was expressed as a percentage [38]. We conducted comparison-adjusted funnel plots using Stata software to examine the publication bias.

## Results

### Study search and study characteristics

**Fig. 1:**
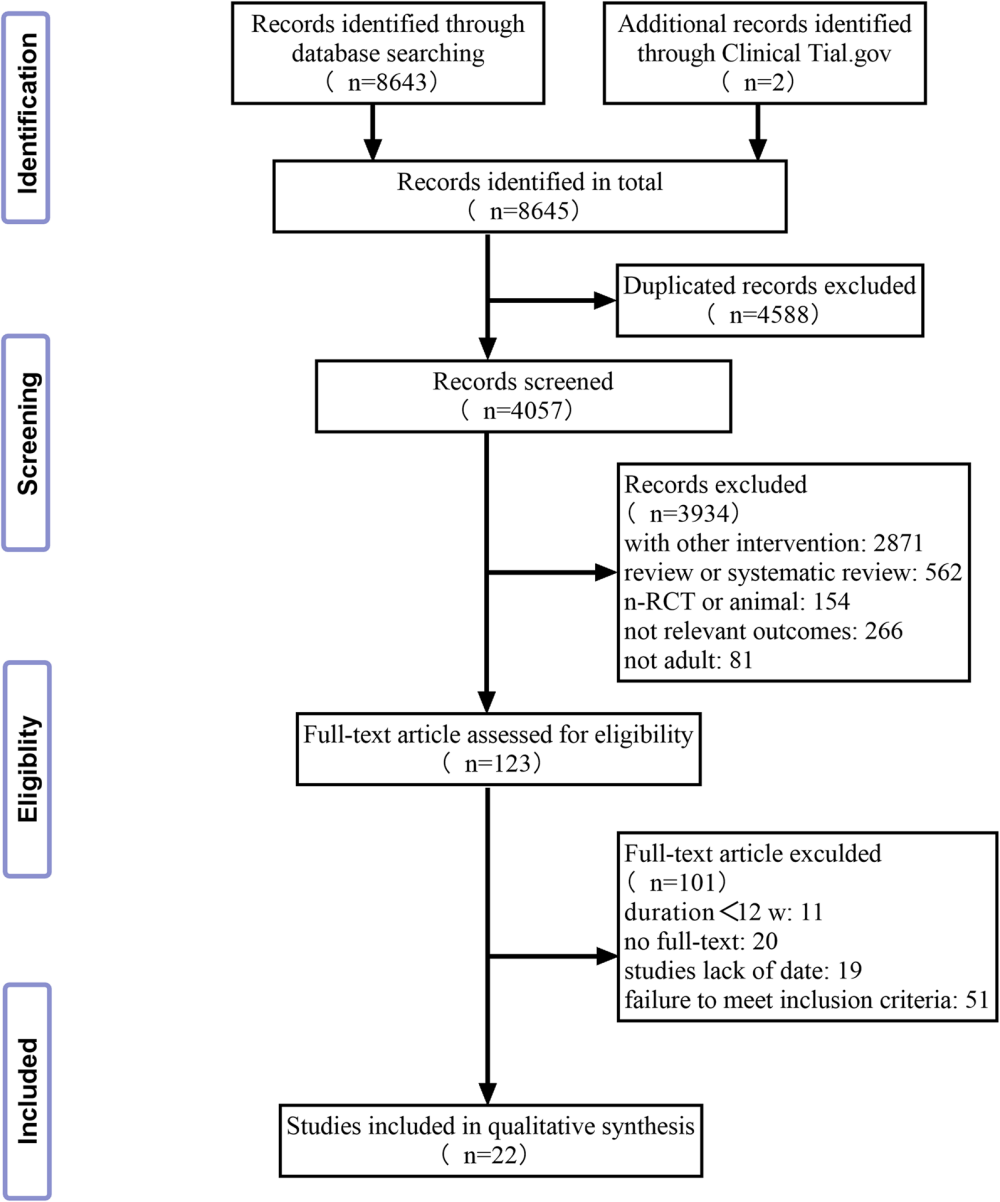
Prisma flow diagram of the study selection process. *RCT* randomized controlled trial

**Fig. 2:**
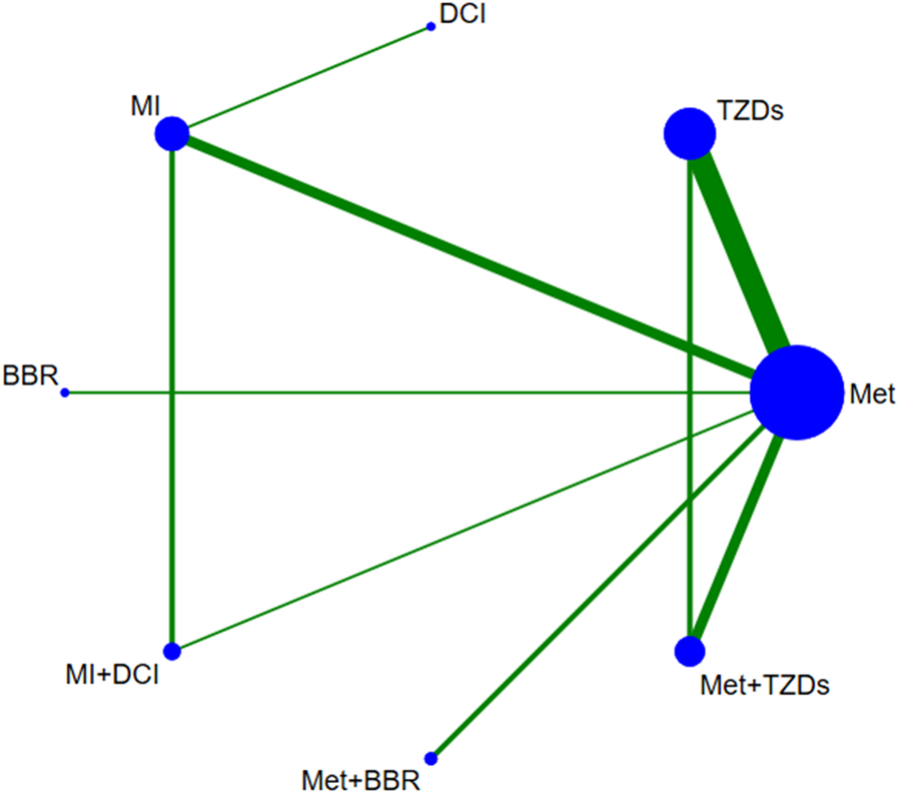
Evidence graph of all agents. The size of the circles is proportional to sample size, and the width of the lines is proportional to the number of trials. *Met* Metformin, *TZDs* thiazolidinediones, *MI* myo-inositol, *DCI* d-chiro-inositol, *BBR* Berberine

A total of 8645 publications were retrieved; of these, 22 RCTs (n = 1079 participants) were included in the present network meta-analysis. A flow chart depicting the literature search process based on PICO is presented in Fig. 1. Overall, two studies evaluated and compared myo-inositol (MI) and myo-inositol + d-chiro-inositol (MI + DCI) combination treatment (72 women), one study compared MI and DCI (50 women), and the remaining RCTs offered a comparison of Met (19 trials; 455 women) and the following interventions: thiazolidinediones (TZDs) (ten trials; 280 women), Met + TZDs (three trials; 89 women), MI (four trials; 110 women), MI + DCI (one trial; 32 women), berberine (BBR) (one trial; 26 women), and Met + BBR (two trials; 70 women). The evidence map for the aforementioned interventions is shown in Fig. 2.

Table 1 shows the characteristics of the included studies. These RCTs were conducted in various countries, published in English or Chinese, and participants were recruited from an outpatient clinic or a hospital. Trials were generally similar with respect to patient baseline characteristics for most outcomes. In summary, a total of 1079 women with PCOS were randomized to receive eight different interventions (Met, TZDs, BBR, MI, DCI, MI + DCI, Met + TZDs, and Met + BBR).

**Fig. 3:**
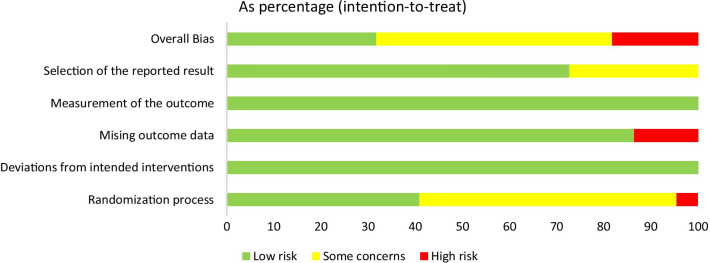
Risk of bias assessment in the RCTs

The risk of bias assessment is shown in Fig. 3. Of the 22 included trials, seven were at low risk of bias across all domains, four studies were at a high risk of bias, and we judged the remaining eleven trials to have some concerns. Overall, concerns regarding the randomization process and missing outcome data were the main cause of potential bias. The comparison-adjusted funnel plot for outcomes appeared minor asymmetry or no publication bias (shown in Additional file 1: Appendix S1.1). In addition, the analyses of inconsistency were identified in the network meta-analysis of triglyceride, and the inconsistency model was fitted in the network meta-analysis, the detailed results of the assessment for inconsistency are shown in Additional file 1: Appendix S1.

### Efficacy outcomes

Table 2A, B summarizes the NMA and TMA results for positive efficacy outcomes. Furthermore, other efficacy outcomes, details of the Forest plots, and SUCRA curves are presented in Additional file 1: Appendix S2.

**Table 2:** (A) NMA and TMA results for menstrual frequency, total testosterone, BMI, and glycometabolism; (B) NMA and TMA results for lipid levels and gastrointestinal adverse events

Outcomes	Studies	Participants	Traditional pairwise meta-analysis (TMA)		Network meta-analysis (NMA)	
			Heterogeneity	Effect estimate (95% CI)	Studies	Effect estimate (95% CI)
(A)						
Menstrual frequency						
TZDs vs Met	6	299	(*P* = 0.002); *I*^*2*^ = 74%	1.17 [0.43, 3.17]	7	1.20 [0.52, 2.76]
TZDs + Met vs Met	3	167	(*P* = 0.88); *I*^*2*^ = 0%	**3.91 [1.75, 8.72]**	3	2.41 [0.75, 7.71]
MI vs Met	1	46	Not applicable	1.11 [0.24, 5.11]	4	0.89 [0.14, 5.56]
MI + DCI vs Met	1	64	Not applicable	11.67 [2.37, 57.36]	3	**14.70 [2.31, 93.58]**
MI + DCI vs MI	2	44	(*P* = 0.38); *I*^*2*^ = 0%	**21.92 [2.99, 160.44]**	3	**16.51 [2.56, 106.64]**
TT						
TZDs vs Met	7	322	(*P* = 0.22); *I*^*2*^ = 27%	**5.27 [0.98, 9.55]**	8	**3.90 [1.10, 6.71]**
TZDs + Met vs Met	4	307	(*P* = 0.44); *I*^*2*^ = 0%	− **3.14 [**− **6.19, **− **0.09]**	4	− **3.66 [**− **6.60, **− **0.72]**
MI vs Met	2	120	(*P* = 0.66); *I*^*2*^ = 0%	0.31 [− 0.69, 1.32]	6	0.37 [− 0.63, 1.37]
MI + DCI vs Met	1	64	Not applicable	− 5.48 [− 10.27, − 0.69]	3	− **6.72 [**− **10.24, **− **3.20]**
BBR + Met vs Met	2	140	(*P* = 1.00); *I*^*2*^ = 0%	− **11.53 [**− **16.91, **− **6.15]**	2	− **11.53 [**− **16.91, **− **6.15]**
MI + DCI vs MI	2	72	(*P* = 0.63); *I*^*2*^ = 0%	− **8.50 [**− **13.60, **− **3.40]**	3	**− 7.09 [− 10.62, − 3.56]**
BMI						
TZDs vs Met	8	322	(*P* = 0.42); *I*^*2*^ = 1%	**1.26 [0.78, 1.75]**	7	**1.23 [0.75, 1.70]**
TZDs + Met vs Met	4	171	(*P* = 0.68); *I*^*2*^ = 0%	− 0.03 [− 1.02, 0.95]	3	0.22 [− 0.71, 1.14]
MI vs Met	5	279	(*P* = 0.47); *I*^*2*^ = 0%	**0.28 [0.06, 0.50]**	8	**0.29 [0.09, 0.49]**
MI + DCI vs Met	/	/	/	/	3	− 0.19 [− 1.61, 1.23]
BBR + Met vs Met	2	140	(*P* = 0.32); *I*^*2*^ = 0%	− **1.85 [**− **2.76, **− **0.94]**	2	− **1.85 [**− **2.76, **− **0.94]**
MI + DCI vs MI	3	80	(*P* = 0.98); *I*^*2*^ = 0%	− 0.48 [− 1.89, 0.93]	3	− 0.48 [− 1.89, 0.93]
FPG						
TZD vs Met	7	272	(*P* < 0.00001); *I*^*2*^ = 86%	− 0.10 [− 0.21, 0.01]	5	− **0.12 [**− **0.21, **− **0.02]**
TZD + Met vs Met	5	307	(*P* = 0.79); *I*^*2*^ = 0%	− 0.05 [− 0.15, 0.06]	5	0.00 [− 0.14, 0.15]
MI vs Met	3	173	(*P* = 0.22); *I*^*2*^ = 34%	− 0.05 [− 0.12, 0.02]	5	− 0.05 [− 0.19, 0.09]
MI + DCI vs Met	1	64	Not applicable	− 0.09 [− 0.17, − 0.01]	6	− 0.18 [− 0.37, 0.02]
MI + DCI vs MI	2	72	(*P* = 0.22); *I*^*2*^ = 35%	− 0.25 [− 0.56, 0.05]	3	− 0.13 [− 0.33, 0.08]
FINS						
TZDs vs Met	8	357	(*P* < 0.00001); *I*^*2*^ = 97%	− 2.50 [− 6.23, 1.23]	7	− 2.38 [− 4.94, 0.19]
TZDs + Met vs Met	4	259	(*P* = 0.19); *I*^*2*^ = 37%	− **1.83 [**− **3.12, **− **0.55]**	4	− 2.56 [− 6.03, 0.92]
MI vs Met	3	173	(*P* = 0.23); *I*^*2*^ = 32%	− 0.22 [− 0.75, 0.32]	5	− 0.40 [− 4.08, 3.27]
MI + DCI vs Met	1	64	Not applicable	− 1.37 [− 1.56, − 1.18]	3	− 1.38 [− 6.14, 3.38]
MI + DCI vs MI	2	72	(*P* = 0.36); *I*^*2*^ = 0%	− 0.76 [− 1.94, 0.42]	3	− 0.98 [− 5.28, 3.33]
HOMA-IR						
TZDs vs Met	6	317	(*P* < 0.00001); *I*^*2*^ = 97%	− **0.92 [**− **1.64, **− **0.19]**	4	− **0.72 [**− **1.11, **− **0.34]**
TZDs + Met vs Met	4	259	(*P* = 0.04); *I*^*2*^ = 65%	− **0.85 [**− **1.21, **− **0.49]**	4	− **0.86 [**− **1.29, **− **0.43]**
MI vs Met	4	219	(*P* = 0.004); *I*^*2*^ = 78%	− 0.20 [− 0.42, 0.01]	6	− 0.28 [− 0.66, 0.10]
MI + DCI vs Met	1	64	Not applicable	− 1.15 [− 1.25, − 1.05]	3	− **0.89 [**− **1.46, **− **0.32]**
BBR + Met vs Met	2	140	(*P* = 0.37); *I*^*2*^ = 0%	− **0.25 [**− **0.36, **− **0.14]**	2	− 0.25 [− 0.81, 0.31]
MI + DCI vs MI	2	72	(*P* = 0.97); *I*^*2*^ = 0%	− 0.39 [− 0.83, 0.06]	3	− **0.61 [**− **1.18, **− **0.05]**
(B)						
TG						
TZDs vs Met	7	261	(*P* = 0.0002); *I*^*2*^ = 77%	− 0.01 [− 0.19, 0.16]	7	− **0.66 [**− **1.00, **− **0.32]**
TZDs + Met vs Met	3	178	(*P* = 0.74); *I*^*2*^ = 0%	− **0.24 [**− **0.43, **− **0.06]**	3	− **0.08 [**− **0.16, **− **0.00]**
MI vs Met	3	173	(*P* = 0.60); *I*^*2*^ = 0%	− 0.03 [− 0.06, 0.00]	3	**0.14 [0.07, 0.21]**
MI + DCI vs Met	1	64	Not applicable	− 0.08 [− 0.16, − 0.00]	4	0.21 [− 0.26, 0.68]
TZDs + Met vs TZDs	2	88	(*P* = 0.10); *I*^*2*^ = 0%	0.15 [− 0.18, 0.48]	3	− **0.51 [**− **0.88, **− **0.14]**
TC						
TZDs vs Met	8	346	(*P* < 0.00001); *I*^*2*^ = 93%	− 0.06 [− 0.41, 0.29]	8	− 0.18 [− 0.46, 0.10]
TZDs + Met vs Met	3	178	(*P* = 0.98); *I*^*2*^ = 0%	− **0.30 [**− **0.53, **− **0.07]**	3	− 0.15 [− 0.57, 0.27]
MI vs Met	3	173	(*P* = 0.67); *I*^*2*^ = 0%	0.03 [− 0.01, 0.07]	3	0.19 [− 0.29, 0.66]
MI + DCI vs Met	1	64	Not applicable	− 0.07 [− 0.16, 0.02]	4	− 0.07 [− 0.69, 0.55]
HDL						
TZDs vs Met	8	346	(*P* < 0.00001); *I*^*2*^ = 96%	0.14 [− 0.03, 0.30]	8	**0.13 [0.03, 0.24]**
TZDs + Met vs Met	4	259	(*P* = 0.53); *I*^*2*^ = 0%	− 0.00 [− 0.09, 0.09]	4	0.05 [− 0.10, 0.20]
MI vs Met	3	173	(*P* = 0.10); *I*^*2*^ = 57%	**0.05 [0.03, 0.07]**	3	0.02 [− 0.14, 0.17]
MI + DCI vs Met	1	64	Not applicable	0.00 [− 0.11, 0.11]	4	0.00 [− 0.28, 0.28]
LDL						
TZDs vs Met	7	261	(*P* = 0.02); *I*^*2*^ = 59%	− 0.11 [− 0.29, 0.08]	7	− **0.19 [**− **0.27, **− **0.11]**
TZDs + Met vs Met	4	259	(*P* = 0.96); *I*^2^ = 0%	− 0.10 [− 0.25, 0.05]	4	− 0.08 [− 0.24, 0.07]
MI vs Met	3	173	(*P* = 0.84); *I*^*2*^ = 0%	0.01 [− 0.03, 0.05]	3	0.01 [− 0.03, 0.05]
MI + DCI vs Met	1	64	Not applicable	− 0.07 [− 0.21, 0.07]	4	− 0.07 [− 0.21, 0.07]
Gastrointestinal adverse events						
TZDs vs Met	6	253	(*P* = 0.88); *I*^*2*^ = 0%	**0.11 [0.03, 0.41]**	6	**0.13 [0.04, 0.46]**
TZDs + Met vs Met	3	237	(*P* = 0.33); *I*^*2*^ = 11%	0.67 [0.23, 1.93]	3	0.76 [0.25, 2.27]
MI vs Met	1	50	Not applicable	0.13 [0.01, 2.58]	1	0.13 [0.01, 2.58]
MI + DCI vs Met	1	64	Not applicable	0.08 [0.00, 1.45]	1	0.08 [0.00, 1.45]
BBR vs Met	1	60	Not applicable	3.10 [0.12, 79.23]	1	3.10 [0.12, 79.23]

Bolded results show statistical significance at the 0.05 level

*Met* Metformin, *TZDs* thiazolidinediones, *MI* myo-inositol, *DCI* d-chiro-inositol, *BBR* berberine, *TT* total testosterone, *BMI* body mass index, *FPG* fasting plasma glucose, *FINS* fasting insulin, *HOMA-IR* Homeostatic Model Assessment of Insulin Resistance, *TG* triglyceride, *TC* total cholesterol, *HDL-C* high density lipoprotein cholesterol, *LDL-C* low density lipoprotein cholesterol

### Menstrual frequency

With respect to improving menstrual frequency, our TMA showed that Met + TZDs (OR 3.91 [95% CI 1.75–8.72]; *P* = 0.88, *I*^*2*^ = 0%) was more efficacious than Met alone, while MI + DCI (OR 21.92 [95% CI 2.99–160.44]; *P* = 0.38, *I*^2^ = 0%) was more efficacious than MI alone. The NMA revealed that MI + DCI was more efficacious than Met (OR 14.70 [95% CI 2.31–93.58]) and MI (OR 16.51 [95% CI 2.56–106.64]). MI + DCI was ranked best at improving menstrual frequency.

### Hyperandrogenism

In terms of reducing TT, the TMA and NMA both revealed that Met + BBR [(TMA: MD − 11.53 [95% CI (− 16.91)–(− 6.15)]; *P* = 1.00, *I*^2^ = 0%; NMA: MD − 11.53 [95% CI (− 17.62)–(− 5.44)]) and Met + TZDs [(TMA: MD − 3.14 [95% CI (− 6.19)–(− 0.09)]; *P* = 0.44, *I*^2^ = 0%; NMA: MD − 3.66 [95% CI (− 6.60)–(− 0.72)] were superior to Met alone. The NMA revealed that MI + DCI was more efficacious than Met, MI, and DCI [MDs ranging from − 6.72 [95% CI (− 10.24)–(− 3.20)] for Met to − 7.70 [95% CI (− 14.90)–(− 0.50)] for DCI]). Treatment with TZDs was less efficacious than Met (MD 3.90 [95% CI 1.10–6.71]). The SUCRA values were as follows: Met + BBR (92.2%), MI + DCI (75.4%), and Met + TZDs (59.4%). No difference was found in comparisons of SHBG, AND, and mF-G scores.

### Obesity

With respect to BMI reduction, the TMA showed that Met + BBR (MD − 1.85 [95% CI (− 2.76)–(− 0.94)]; *P* = 0.32, *I*^2^ = 0%) was superior to Met monotherapy, whereas the NMA revealed that Met + BBR was more efficacious than Met, MI, TZDs, and Met + TZDs (MDs ranging from − 1.85 [95% CI (− 2.76)–(− 0.94)] for Met to − 3.08 [95% CI (− 4.10)–(− 2.05)] for TZDs). Met + BBR ranked best among all the treatments at reducing BMI. However, both the TMA and NMA revealed that there was no significant difference between the groups with respect to WHR reduction.

### Glycometabolism

With respect to lowering FPG, the TMA revealed that there were no significant differences between each of the investigated agents. The NMA revealed that TZDs (MD − 0.21 [95% CI (− 0.21)–(− 0.02)]) were more efficacious than Met. Additionally, with respect to lowering FINS, the TMA revealed that Met + TZDs (MD − 1.83 [95% CI (− 3.12)–(− 0.55)]; *P* = 0.19, *I*^2^ = 37%) was more efficacious than Met alone. The NMA did not reveal any significant differences among the groups.

With respect to decrease in HOMA-IR, the TMA showed that TZDs (MD − 0.92 [95% CI (− 1.64)–(− 0.19)]; *P* < 0.00001, *I*^2^ = 97%), Met + TZDs (MD − 0.85 [95% CI (− 1.21)–(− 0.49)]; *P* = 0.04, *I*^2^ = 65%), and Met + BBR (MD − 0.25 [95% CI (− 0.36)–(− 0.14)]; *P* = 0.37, *I*^2^ = 0%) were more efficacious than Met alone. The NMA revealed that TZDs, Met + TZDs, and MI + DCI were all superior to Met (MDs ranging from − 0.72 [95% CI (− 1.11)–(− 0.34)] for TZDs to − 0.89 [95% CI (− 1.46)–(− 0.32)] for MI + DCI), with MI + DCI being ranked the best with a SUCRA value of 80.8%. The SUCRA values for the other agents were as follows: 79.9% and 68.6% for Met + TZDs and TZDs, respectively.

### Lipid levels

Parameters assessed for improvement in blood lipids included TG, TC, HDL-C, and LDL-C. In terms of reducing TG and TC levels, the TMA revealed that Met + TZDs (TG: MD − 0.24 [95% CI (− 0.43)–(− 0.06)]; *P* = 0.74, *I*^2^ = 0%; TC: MD − 0.30 [95% CI (− 0.53)–(− 0.07)]; *P* = 0.98, *I*^2^ = 0%) was more efficacious than Met alone. In terms of reducing levels of TG, the NMA showed that Met + TZDs was superior to Met (MD − 0.08 [95% CI (− 0.16)–(0.00)]) and TZDs (MD − 0.51 [95% CI (− 0.88)–(− 0.14)]), and was the best intervention among treatments. The NMA also showed that BBR (MD − 0.03 [95% CI (− 0.06)–(0.00)]) was more efficacious than Met. However, for TC no significant differences were found in the NMA.

Furthermore, the TMA also suggested that MI (MD 0.05 [95% CI 0.03–0.07]; *P* = 0.10, *I*^2^ = 57%) was associated with higher HDL-C than Met. The NMA revealed that treatment with TZDs was superior to Met in increasing HDL-C (MD 0.13 [95% CI 0.03–0.24]) and decreasing LDL-C (MD − 0.19 [95% CI (− 0.27)–(− 0.11)]) and was the best intervention among the different treatments.

### Adverse events

In terms of the frequency of gastrointestinal adverse events during treatment, both TMA and NMA revealed that treatment with TZDs (TMA: OR 0.11 [95% CI 0.03–0.41]; *P* = 0.88, *I*^2^ = 0%; NMA: OR 0.13 [95% CI 0.04–0.46]) was inferior to Met. With respect to the incidence of peripheral edema, the TMA revealed that TZDs were more frequent than Met (OR 67.89 [95% CI 3.96, 1163.28]; *P&I*^2^ NA), no significant differences were found in the NMA. Furthermore, both the TMA and NMA did not show any significant differences between the different treatment regimens for the incidence rate of muscle spasms, while no drug increased odds of transaminase abnormalities. Of note, these results should be interpreted with caution since results for MI, MI + DCI, and BBR were based on a single trial, and in some of the trials the description of adverse events was subjective and unclear.

## Discussion

To our knowledge, this is the first report on an NMA used to assess the efficacy and safety of oral insulin sensitizers (metformin, thiazolidinediones, inositol, and berberine) as an adjunct therapy to improve irregular menses, hyperandrogenism, and glucolipid metabolism abnormalities in women with PCOS. The results obtained are based on 22 trials that included 1079 women, randomly assigned to eight different interventions. Overall, treatment with MI + DCI was associated with the best improvement in menstrual frequency. MI + DCI, Met + TZDs, and Met + BBR were superior to Met for TT reduction, while MI + DCI, Met + TZDs, and TZDs significantly lowered HOMA-IR than Met alone. TZDs were superior to Met in decreasing FPG, TG, LDL-C levels, and increasing HDL-C level, while Met + TZDs was associated with lower TG levels compared to Met and TZDs monotherapy. Furthermore, Met + BBR was more efficacious than Met alone in reducing BMI.

Metformin is a classic insulin sensitizer, that inhibits hepatic glucose production, thereby decreasing glucose levels [39]. Many studies have already demonstrated its ability to improve menstruation frequency, reduce androgen excess, and decrease insulin resistance in PCOS. However, it also often associated with gastrointestinal side effects, such as diarrhea, nausea, and abdominal discomfort [40]. In the current study, in four of the RCTs, a total of 12 women withdrew due to intolerance rising from gastrointestinal side effects [14, 19, 23, 26]. There were other reports of mild stomach discomfort that resolved spontaneously within a few weeks [13, 15, 16, 18, 20, 21, 28].

Thiazolidinediones (TZDs) decrease hepatic and peripheral insulin resistance directly through activation of the nuclear hormone receptor PPARγ and have a well-documented effect of improving hyperglycemia and dyslipidemia. TZDs also improve the menstrual cycle and ovulation and reduce androgen levels in women with PCOS [41, 42]. However, these clinical benefits have largely been ignored due to safety issues and side effects, such as weight gain, peripheral edema, and even heart failure [43]. In our study, one trial with pioglitazone showed 40% of women with mild peripheral edema and 11% with muscle spasms [16], although other adverse events were not found and gastrointestinal adverse events were less frequent with TZDs than Met. Furthermore, compared with that by Met, TZDs had a more beneficial effect on improving glucolipid metabolism; our NMA revealed that treatment with TZDs was associated with lower FPG, TG, LDL-C levels, and higher HDL-C level. The SUCRA analysis revealed that treatment with TZDs was the best (among these treatments) for decreasing LDL-C and increasing HDL-C, indicating its potential ability to reduce a patients’ risk of cardiovascular diseases in PCOS.

Our NMA revealed that the combination of Met + TZDs was more effective than Met alone at improving menstruation frequency, and reducing TT, HOMA-IR, and TG. The SUCRA value showed that Met + TZDs was the best intervention for reducing TG level. The TMA also demonstrated that Met + TZDs was superior to Met monotherapy at reducing FINS and TC level. Our previous NMA in overweight women with PCOS revealed that Met + TZDs was superior to Met in recovering menstrual function, whereas there were no evident differences in TT and FINS [44]. Different inclusion criteria might explain these discrepancies. Additionally, three RCTs reported gastrointestinal discomfort in the Met + TZDs group [20, 21, 23], which was not significantly different than the Met alone group.

Inositol acts as a second messenger with insulin-like functions and is safe and well-tolerated [45]. The two most common isomers of inositol are MI, which has been shown to significantly improve ovulatory function [46], and DCI, which is able to reduce peripheral insulin resistance in patients with PCOS [47]. Our NMA is in line with other pairwise meta-analyses, showing that there is no apparent benefit of MI or DCI alone compared with Met [48–50]. Recent studies have proposed that a combination of both MI and DCI, at a plasma ratio of 40:1, can restore normal hormonal function quicker than MI or DCI alone [51]. This is the first report on the analysis of efficacy of Met and MI + DCI in women with PCOS; it revealed that among all of the agents investigated here MI + DCI appeared to be the best intervention for restoring regular menses and decreasing HOMA-IR, while the combination was also able to reduce TT better than Met. No side effects have been described in clinical studies examining the effect of inositol [26, 44].

Berberine (BBR), a natural isoquinoline alkaloid, has been studied in various randomized clinical trials in patients with PCOS and has been shown to be safe and promising for decreasing insulin resistance, lowering blood lipids, and restoring ovulation [52–55]. In the only previous pairwise meta-analysis that compared Met, BBR, and their combination in insulin resistant patients with PCOS, there were no significant differences between the different treatment groups [56]. In the current study, only one trial that examined BBR and Met was included. As seen from the NMA, BBR was superior to Met in reducing TG. Met + BBR was associated with a greater reduction in TT and BMI than that by Met alone and was the best intervention among the investigated agents tested here. These findings should be interpreted with caution since they are based on data from only two head-to-head trials of low quality [33, 34]. Further high-quality trials to verify these potential favorable effects in PCOS are therefore required. One person in the berberine group withdrew due to gastrointestinal side effects [32].

## Strengths and limitations

Our findings reflect the comparative efficacy and safety of monotherapy versus a combination of different oral insulin sensitizers (metformin, thiazolidinediones, inositol, and berberine) in the treatment of PCOS. Previous related meta-analyses have mainly been of pairwise design that focused on individual agents and have given inconsistent results, our NMA incorporated both direct and indirect comparisons of interventions into a single analysis and provided a ranking of the available interventions. Through a comprehensive search and a rigorous review of the design of the randomized clinical trials, we aimed to reduce the likelihood of selection bias. We focused on the analysis of menstrual and ovulation abnormalities, hyperandrogenism, obesity, glucolipid metabolism, and adverse events in women with PCOS to provide a comprehensive reference for its clinical treatment.

However, the present study still has some limitations that must be noted. First, very few literature reports met the inclusion criteria of treatment without contraceptives and/or intervention of ovulation inducing drugs to effectively verify the independent effects of drugs on efficacy. Second, there were markedly different dosages, durations of treatment, and small sample sizes that could contribute to sample bias, selection bias, and high heterogeneity. Third, some of trials did not have a specific analysis of women who dropped out or missed a follow-up.

## Conclusions

In conclusion, for women with PCOS, MI combined with DCI and Met combined with TZDs appear to be superior than Met alone in improving insulin resistance and decreasing total testosterone. MI combined with DCI appears to be particularly efficacious in menstrual recovery. TZDs and TZDs combined with Met seem to offer the additional advantage of improving lipid metabolism. However, our findings are limited by the small number and low quality of available studies, and therefore more rigorous and high-level RCTs are needed to further guide the clinical management of women with PCOS, especially those with insulin resistance.

## Supplementary Information

**Additional file 1: Appendix S1.** Details on study methods.

## Data Availability

The datasets supporting the conclusions of this article are included within the article and its additional file.

## Abbreviations

PCOS: Polycystic ovary syndrome
Met: Metformin
TZDs: Thiazolidinediones
MI: Myo-inositol
DCI: D-chiro-inositol
BBR: Berberine
TT: Total testosterone
SHBG: Sex hormone binding globulin
AND: Androstenedione
mF-Gscore: Modified Ferriman–Gallwey score
BMI: Body mass index
WHR: Waist–hip ratio
FPG: Fasting plasma glucose
FINS: Fasting insulin
HOMA-IR: Homeostatic Model Assessment of Insulin Resistance
TG: Triglyceride
TC: Total cholesterol
HDL-C: High density lipoprotein cholesterol
LDL-C: Low density lipoprotein cholesterol
