# Supplementary material for: Comparative efficacy of oral insulin sensitizers metformin, thiazolidinediones, inositol, and berberine in improving endocrine and metabolic profiles in women with PCOS: a network meta-analysis
Source: Reprod Health. 2021 Aug 18;18:171. doi: 10.1186/s12978-021-01207-7 (PMC8371888; doi:10.1186/s12978-021-01207-7)
Supplement: Supplementary file 1 — Additional file 1: Appendix S1. Details on study methods. [file 12978_2021_1207_MOESM1_ESM.docx]

**Appendix S1**

1.1 Comparison-adjusted funnel plot for all outcomes^1^


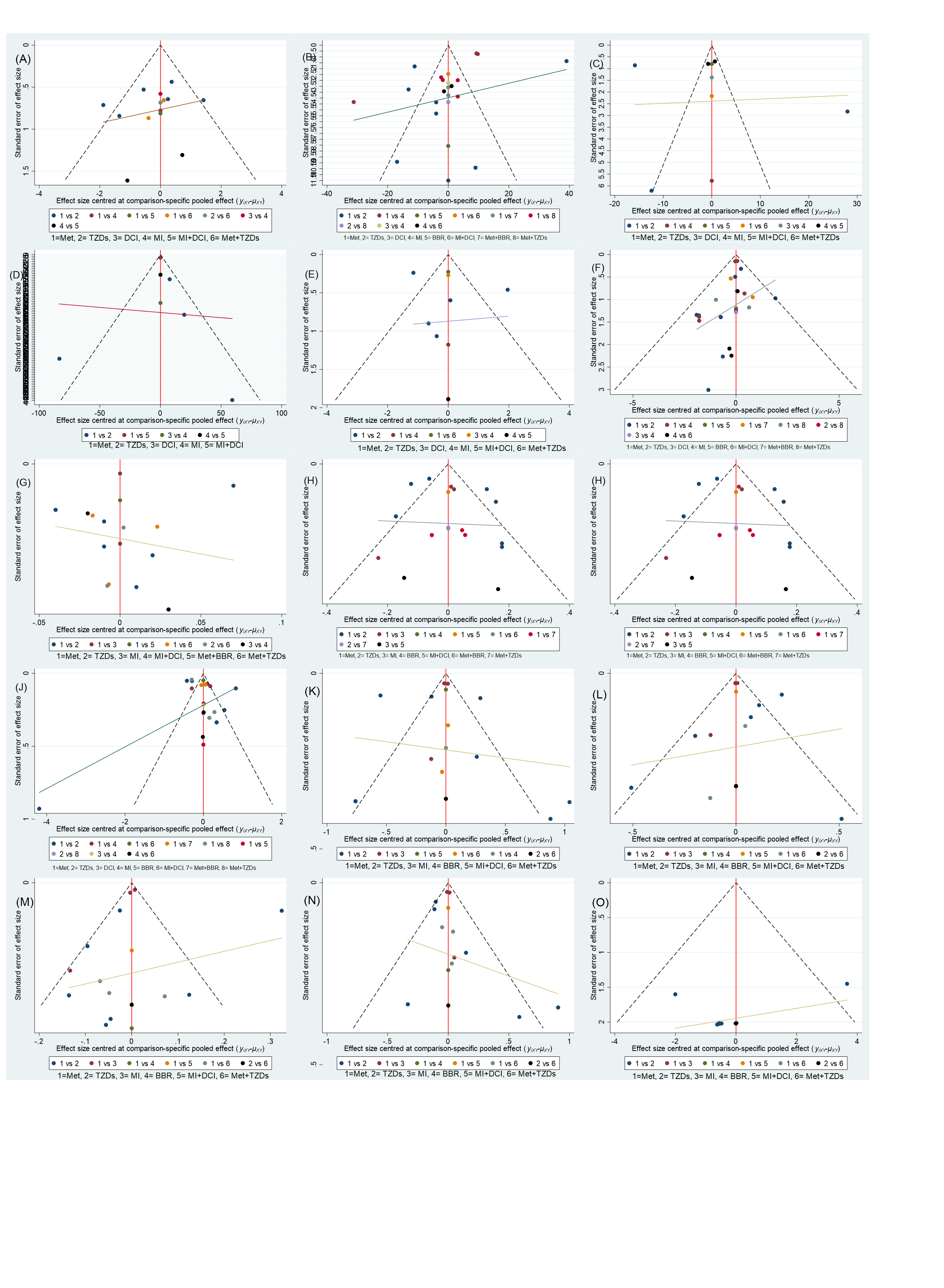


^1^(A) Menstrual frequency; (B) TT, total testosterone; (C) SHBG, sex hormone binding globulin; (D) AND, androstenedione; (E) mF-G score, modified Ferriman-Gallwey score; (F) BMI, body mass index; (G) WHR, waist-hip ratio; (H) FPG, fasting plasma glucose; (I) FINS, fasting insulin; (J) HOMA-IR, Homeostatic Model Assessment of Insulin Resistance; (K) TC, total cholesterol; (L) TG, triglyceride; (M) HDL-C, high density lipoprotein cholesterol; (N) LDL-C, low density lipoprotein cholesterol; (O) Gastrointestinal adverse events.

Abbreviations: Met, Metformin; TZDs, Thiazolidinediones; MI, Myo-inositol; DCI, D-chiro-inositol; BBR, Berberine;

1.2 Network plot of eligible comparisons for all outcomes^1^


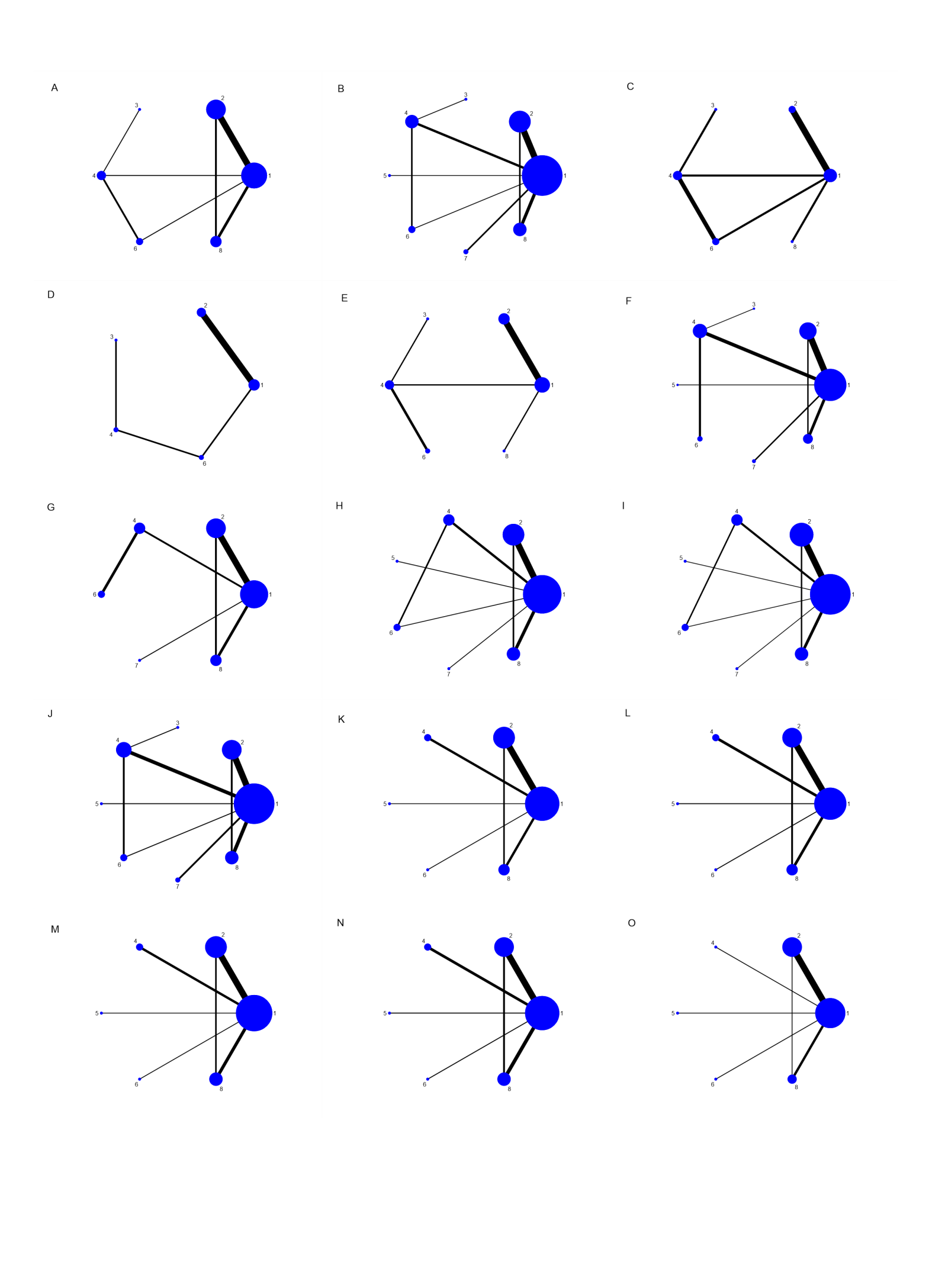


^1^(A) Menstrual frequency; (B) TT, total testosterone; (C) SHBG, sex hormone binding globulin; (D) AND, androstenedione; (E) mF-G score, modified Ferriman-Gallwey score; (F) BMI, body mass index; (G) WHR, waist-hip ratio; (H) FPG, fasting plasma glucose; (I) FINS, fasting insulin; (J) HOMA-IR, Homeostatic Model Assessment of Insulin Resistance; (K) TC, total cholesterol; (L) TG, triglyceride; (M) HDL-C, high density lipoprotein cholesterol; (N) LDL-C, low density lipoprotein cholesterol; (O) Gastrointestinal adverse events. (1) Met, Metformin; (2) TZDs, Thiazolidinediones; (3) DCI, D-chiro-inositol; (4) MI, Myo-inositol; (5) BBR, Berberine; (6) MI+DCI; (7) Met+BBR; (8) Met+TZDs；

1.3 Summary of the results of Cochrane risk of bias ROB2.

| **Study ID** | **Experimental** | **Comparator** | **Randomization process** | **Deviations from intended interventions** | **Missing outcome data** | **Measurement of the outcome** | **Selection of the reported result** | **Overall** |  |  |
| --- | --- | --- | --- | --- | --- | --- | --- | --- | --- | --- |
| Ahmad 2008 | TZDs | Met | 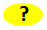 | 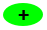 | 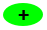 | 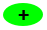 | 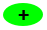 | 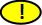 | 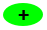 | Low risk |
| Ortega 2005 | TZDs | Met | 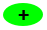 | 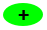 | 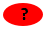 | 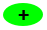 | 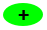 | 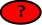 | 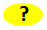 | Some concerns |
| Pizzo 2014 | DCI | MI | 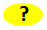 | 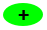 | 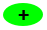 | 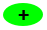 | 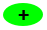 | 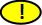 | 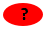 | High risk |
| Sangeeta 2012 | TZDs | Met | 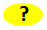 | 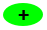 | 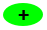 | 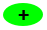 | 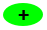 | 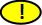 |  |  |
| Sohrevardi 2016 | TZDs, Met+TZDs | Met | 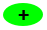 | 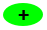 | 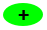 | 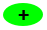 | 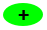 | 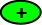 |  |  |
| Wang L 2011 | Met+BBR | Mer | 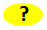 | 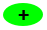 | 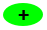 | 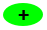 | 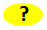 | 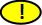 |  |  |
| Wang P 2016 | Met+BBR | Met | 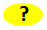 | 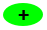 | 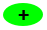 | 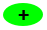 | 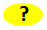 | 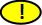 |  |  |
| Wang X 2014 | Met+TZDs | Met | 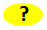 | 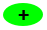 | 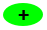 | 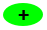 | 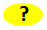 | 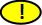 |  |  |
| Yilmaz 2005 | TZDs | Met | 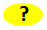 | 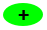 | 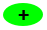 | 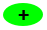 | 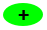 | 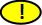 |  |  |
| Zeng 2020 | Met+TZDs | Met | 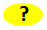 | 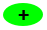 | 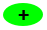 | 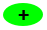 | 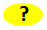 | 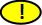 |  |  |
| Shokrpour 2019 | MI | Met | 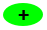 | 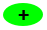 | 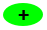 | 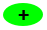 | 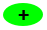 | 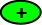 |  |  |
| Du 2018 | MI+DCI | Met | 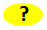 | 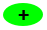 | 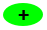 | 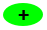 | 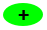 | 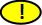 |  |  |
| Jamilian 2017 | MI | Met | 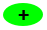 | 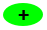 | 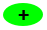 | 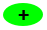 | 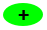 | 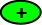 |  |  |
| Fruzzetti 2016 | MI | Met | 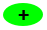 | 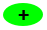 | 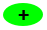 | 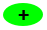 | 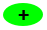 | 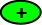 |  |  |
| Donne 2019 | MI+DCI | MI | 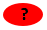 | 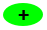 | 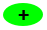 | 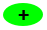 | 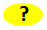 | 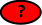 |  |  |
| Jensterl 2008 | TZDs | Met | 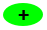 | 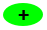 | 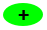 | 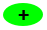 | 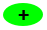 |  |  |  |
| Li 2017 | BBR | Met |  |  |  |  |  |  |  |  |
| Liang 2019 | TZDs, Met+TZDs | Met |  |  |  |  |  |  |  |  |
| Mohiyididden 2013 | TZDs | Met |  |  |  |  |  |  |  |  |
| Naka 2011 | TZDs | Met |  |  |  |  |  |  |  |  |
| Nehra 2017 | MI | Met |  |  |  |  |  |  |  |  |
| Nordio 2012 | MI+DCI | MI |  |  |  |  |  |  |  |  |

Abbreviations: Met, Metformin; TZDs, Thiazolidinediones; MI, Myo-inositol; DCI, D-chiro-inositol; BBR, Berberine;

1.4 Inconsistency Models for the assessment of global inconsistency^1^

| Outcomes | Testing for inconsistency |
| --- | --- |
|  | *P* |
| Menstrual frequency | 0.32 |
| Gastrointestinal adverse events | 0.97 |
| total testosterone | 0.84 |
| androstenedione | 0.90 |
| androstenedione | no source of inconsistency |
| modified Ferriman-Gallwey score | no source of inconsistency |
| body mass index | 0.25 |
| waist-hip ratio | 0.75 |
| fasting plasma glucose | 0.28 |
| fasting insulin | 0.97 |
| Homeostatic Model Assessment of Insulin Resistance | 0.68 |
| total cholesterol | 0.25 |
| triglyceride | 0.04 |
| high density lipoprotein cholesterol | 0.29 |
| low density lipoprotein cholesterol | 0.68 |

^1^*P* for the assessment of inconsistency. *P* <0.05 was considered to indicate a significant inconsistency existed in the network meta-analysis.

1.5 Node-splitting method for the assessment of local inconsistency for all efficacy outcomes^1^

| **FPG** |  | Direct | |  | Indirect | |  | Difference | | *P* | *tau* |
| --- | --- | --- | --- | --- | --- | --- | --- | --- | --- | --- | --- |
| Comparisons |  | Coefficient | SE |  | Coefficient | SE |  | Coefficient | SE |  |  |
| Met vs. TZDs |  | -0.10 | 0.05 |  | -0.11 | 0.35 |  | 0.01 | 0.35 | 0.98 | 0.12 |
| Met vs. MI |  | -0.08 | 0.07 |  | 0.17 | 0.19 |  | -0.24 | 0.20 | 0.23 | 0.11 |
| Met vs. BBR |  | - | - |  | - | - |  | - | - | - | - |
| Met vs. MI+DCI |  | -0.09 | 0.12 |  | -0.33 | 0.16 |  | 0.24 | 0.20 | 0.23 | 0.11 |
| Met vs. Met+BBR |  | - | - |  | - | - |  | - | - | - | - |
| Met vs. Met+TZDs |  | -0.03 | 0.08 |  | 0.35 | 0.25 |  | -0.38 | 0.26 | 0.14 | 0.11 |
| TZDs vs. Met+TZDs |  | 0.22 | 0.14 |  | 0.04 | 0.11 |  | 0.18 | 0.18 | 0.33 | 0.11 |
| MI vs. MI+DCI |  | -0.26 | 0.15 |  | -0.01 | 0.14 |  | -0.24 | 0.20 | 0.23 | 0.11 |

| **FINS** |  | Direct | |  | Indirect | |  | Difference | | *P* | *tau* |
| --- | --- | --- | --- | --- | --- | --- | --- | --- | --- | --- | --- |
| Comparisons |  | Coefficient | SE |  | Coefficient | SE |  | Coefficient | SE |  |  |
| Met vs. TZDs |  | -2.26 | 1.25 |  | 1.12 | 6.97 |  | -3.38 | 7.08 | 0.63 | 3.56 |
| Met vs. MI |  | -0.41 | 2.10 |  | -0.38 | 4.45 |  | -0.03 | 4.92 | 1.00 | 3.60 |
| Met vs. BBR |  | - | - |  | - | - |  | - | - | - | - |
| Met vs. MI+DCI |  | -1.37 | 3.60 |  | -1.39 | 3.36 |  | 0.02 | 4.92 | 1.00 | 3.60 |
| Met vs. Met+BBR |  | - | - |  | - | - |  | - | - | - | - |
| Met vs. Met+TZDs |  | -2.26 | 1.88 |  | -4.43 | 5.51 |  | 2.17 | 5.84 | 0.71 | 3.56 |
| TZDs vs. Met+TZDs |  | -1.30 | 2.69 |  | 0.70 | 2.82 |  | -2.00 | 3.90 | 0.61 | 3.55 |
| MI vs. MI+DCI |  | -0.98 | 2.63 |  | -0.96 | 4.16 |  | -0.02 | 4.92 | 1.00 | 3.60 |

| **HOMA-IR** |  | Direct | |  | Indirect | |  | Difference | | *P* | *tau* |
| --- | --- | --- | --- | --- | --- | --- | --- | --- | --- | --- | --- |
| Comparisons |  | Coefficient | SE |  | Coefficient | SE |  | Coefficient | SE |  |  |
| Met vs. TZDs |  | -0.81 | 0.19 |  | -0.89 | 0.90 |  | 0.09 | 0.92 | 0.92 | 0.42 |
| Met vs. MI |  | -0.21 | 0.21 |  | -0.76 | 0.55 |  | 0.55 | 0.59 | 0.35 | 0.40 |
| Met vs. BBR |  | - | - |  | - | - |  | - | - | - | - |
| Met vs. MI+DCI |  | -1.15 | 0.41 |  | -0.60 | 0.43 |  | -0.55 | 0.59 | 0.35 | 0.40 |
| Met vs. Met+BBR |  | - | - |  | - | - |  | - | - | - | - |
| Met vs. Met+TZDs |  | -0.82 | 0.25 |  | -1.49 | 0.79 |  | 0.68 | 0.84 | 0.42 | 0.43 |
| TZDs vs. Met+TZDs |  | -0.19 | 0.36 |  | 0.06 | 0.40 |  | -0.25 | 0.54 | 0.64 | 0.43 |
| DCI vs. MI |  | 0.14 | 0.46 |  | -0.70 | 200.10 |  | 0.84 | 200.10 | 1.00 | 0.40 |
| MI vs. MI+DCI |  | -0.39 | 0.38 |  | -0.94 | 0.46 |  | 0.55 | 0.59 | 0.35 | 0.40 |

| **BMI** |  | Direct | |  | Indirect | |  | Difference | | *P* | *tau* |
| --- | --- | --- | --- | --- | --- | --- | --- | --- | --- | --- | --- |
| Comparisons |  | Coefficient | SE |  | Coefficient | SE |  | Coefficient | SE |  |  |
| Met vs. TZDs |  | 1.21 | 0.24 |  | -1.52 | 1.88 |  | 2.73 | 1.89 | 0.15 | 0.00 |
| Met vs. MI |  | 0.29 | 0.10 |  | 0.21 | 50.19 |  | 0.08 | 50.19 | 1.00 | 0.00 |
| Met vs. BBR |  | - | - |  | - | - |  | - | - | - | - |
| Met vs. Met+BBR |  | - | - |  | - | - |  | - | - | - | - |
| Met vs. Met+TZDs |  | -0.03 | 0.50 |  | 2.49 | 1.73 |  | -2.52 | 1.84 | 0.17 | 0.00 |
| TZDs vs. Met+TZDs |  | 0.21 | 0.87 |  | -1.64 | 0.65 |  | 1.85 | 1.11 | 0.10 | 0.00 |
| DCI vs. MI |  | 0.83 | 1.28 |  | -0.25 | 200.03 |  | 1.08 | 200.04 | 1.00 | 0.00 |
| MI vs. MI+DCI |  | -0.48 | 0.72 |  | -1.06 | 115.44 |  | 0.58 | 115.44 | 1.00 | 0.00 |

| **WHR** |  | Direct | |  | Indirect | |  | Difference | | *P* | *tau* |
| --- | --- | --- | --- | --- | --- | --- | --- | --- | --- | --- | --- |
| Comparisons |  | Coefficient | SE |  | Coefficient | SE |  | Coefficient | SE |  |  |
| Met vs. TZDs |  | -0.01 | 0.01 |  | 0.06 | 0.09 |  | -0.07 | 0.09 | 0.43 | 0.03 |
| Met vs. MI |  | -0.01 | 0.03 |  | 0.00 | 55.09 |  | -0.01 | 55.09 | 1.00 | 0.03 |
| Met vs. Met+BBR |  | - | - |  | - | - |  | - | - | - | - |
| Met vs. Met+TZDs |  | 0.01 | 0.02 |  | 0.00 | 0.06 |  | 0.01 | 0.06 | 0.91 | 0.03 |
| TZDs vs. Met+TZDs |  | 0.01 | 0.03 |  | 0.03 | 0.04 |  | -0.02 | 0.05 | 0.63 | 0.03 |
| MI vs. MI+DCI |  | 0.03 | 0.02 |  | 0.04 | 89.16 |  | -0.02 | 89.16 | 1.00 | 0.03 |

| **TC** |  | Direct | |  | Indirect | |  | Difference | | *P* | *tau* |
| --- | --- | --- | --- | --- | --- | --- | --- | --- | --- | --- | --- |
| Comparisons |  | Coefficient | SE |  | Coefficient | SE |  | Coefficient | SE |  |  |
| Met vs. TZDs |  | 0.02 | 0.19 |  | -0.59 | 1.31 |  | 0.62 | 1.32 | 0.64 | 0.44 |
| Met vs. MI |  | - | - |  | - | - |  | - | - | - | - |
| Met vs. BBR |  | - | - |  | - | - |  | - | - | - | - |
| Met vs. MI+DCI |  | - | - |  | - | - |  | - | - | - | - |
| Met vs. Met+TZDs |  | -0.30 | 0.31 |  | 1.52 | 1.00 |  | -1.82 | 1.05 | 0.08 | 0.38 |
| TZDs vs. Met+TZDs |  | 0.44 | 0.54 |  | -0.54 | 0.45 |  | 0.98 | 0.71 | 0.17 | 0.40 |

| **TG** |  | Direct | |  | Indirect | |  | Difference | | *P* | *tau* |
| --- | --- | --- | --- | --- | --- | --- | --- | --- | --- | --- | --- |
| Comparisons |  | Coefficient | SE |  | Coefficient | SE |  | Coefficient | SE |  |  |
| Met vs. TZDs |  | 0.06 | 0.07 |  | -0.28 | 0.57 |  | 0.34 | 0.58 | 0.55 | 0.11 |
| Met vs. MI |  | - | - |  | - | - |  | - | - | - | - |
| Met vs. BBR |  | - | - |  | - | - |  | - | - | - | - |
| Met vs. MI+DCI |  | - | - |  | - | - |  | - | - | - | - |
| Met vs. Met+TZDs |  | -0.23 | 0.10 |  | 0.95 | 0.41 |  | -1.17 | 0.42 | 0.01 | 0.00 |
| TZDs vs. Met+TZDs |  | 0.19 | 0.25 |  | -0.36 | 0.14 |  | 0.54 | 0.29 | 0.06 | 0.07 |

| **HDL** |  | Direct | |  | Indirect | |  | Difference | | *P* | *tau* |
| --- | --- | --- | --- | --- | --- | --- | --- | --- | --- | --- | --- |
| Comparisons |  | Coefficient | SE |  | Coefficient | SE |  | Coefficient | SE |  |  |
| Met vs. TZDs |  | 0.16 | 0.06 |  | -0.25 | 0.36 |  | 0.41 | 0.37 | 0.26 | 0.13 |
| Met vs. MI |  | - | - |  | - | - |  | - | - | - | - |
| Met vs. BBR |  | - | - |  | - | - |  | - | - | - | - |
| Met vs. MI+DCI |  | - | - |  | - | - |  | - | - | - | - |
| Met vs. Met+TZDs |  | -0.02 | 0.09 |  | 0.44 | 0.30 |  | -0.46 | 0.31 | 0.15 | 0.13 |
| TZDs vs. Met+TZDs |  | 0.07 | 0.16 |  | -0.25 | 0.12 |  | 0.32 | 0.20 | 0.11 | 0.13 |

| **LDL** |  | Direct | |  | Indirect | |  | Difference | | *P* | *tau* |
| --- | --- | --- | --- | --- | --- | --- | --- | --- | --- | --- | --- |
| Comparisons |  | Coefficient | SE |  | Coefficient | SE |  | Coefficient | SE |  |  |
| Met vs. TZDs |  | -0.18 | 0.04 |  | -0.37 | 0.46 |  | 0.19 | 0.46 | 0.68 | 0.00 |
| Met vs. MI |  | - | - |  | - | - |  | - | - | - | - |
| Met vs. BBR |  | - | - |  | - | - |  | - | - | - | - |
| Met vs. MI+DCI |  | - | - |  | - | - |  | - | - | - | - |
| Met vs. Met+TZDs |  | -0.10 | 0.08 |  | 0.45 | 0.64 |  | -0.55 | 0.64 | 0.39 | 0.00 |
| TZDs vs. Met+TZDs |  | 0.31 | 0.31 |  | 0.06 | 0.10 |  | 0.24 | 0.33 | 0.46 | 0.00 |

| **TT** |  | Direct | |  | Indirect | |  | Difference | | *P* | *tau* |
| --- | --- | --- | --- | --- | --- | --- | --- | --- | --- | --- | --- |
| Comparisons |  | Coefficient | SE |  | Coefficient | SE |  | Coefficient | SE |  |  |
| Met vs. TZDs |  | 14.35 | 5.99 |  | -0.27 | 29.77 |  | 14.62 | 30.32 | 0.63 | 15.56 |
| Met vs. MI |  | -12.42 | 9.03 |  | 2.86 | 18.40 |  | -15.28 | 20.50 | 0.46 | 15.39 |
| Met vs. BBR |  | - | - |  | - | - |  | - | - | - | - |
| Met vs. MI+DCI |  | -5.48 | 15.61 |  | -20.64 | 14.25 |  | 15.16 | 21.13 | 0.47 | 15.41 |
| Met vs. Met+BBR |  | - | - |  | - | - |  | - | - | - | - |
| Met vs. Met+TZDs |  | -2.64 | 7.90 |  | 11.90 | 24.40 |  | -14.54 | 25.69 | 0.57 | 15.44 |
| TZDs vs. Met+TZDs |  | -10.01 | 11.41 |  | -21.23 | 12.67 |  | 11.23 | 17.11 | 0.51 | 15.40 |
| DCI vs. MI |  | -0.61 | 15.48 |  | -18.39 | 202.37 |  | 17.78 | 203.02 | 0.93 | 15.15 |
| MI vs. MI+DCI |  | -8.64 | 11.21 |  | 6.92 | 17.99 |  | -15.57 | 21.20 | 0.46 | 15.42 |

| **SHBG** |  | Direct | |  | Indirect | |  | Difference | | *P* | *tau* |
| --- | --- | --- | --- | --- | --- | --- | --- | --- | --- | --- | --- |
| Comparisons |  | Coefficient | SE |  | Coefficient | SE |  | Coefficient | SE |  |  |
| Met vs. TZDs |  | - | - |  | - | - |  | - | - | - | - |
| Met vs. MI |  | 5.20 | 19.87 |  | 0.84 | 21.97 |  | 4.36 | 29.62 | 0.88 | 19.01 |
| Met vs. MI+DCI |  | 2.20 | 19.07 |  | 5.92 | 22.89 |  | -3.72 | 29.79 | 0.90 | 19.06 |
| Met vs. Met+TZDs |  | - | - |  | - | - |  | - | - | - | - |
| DCI vs. MI |  | 1.15 | 16.95 |  | 5.41 | 203.89 |  | -4.26 | 204.60 | 0.98 | 16.89 |
| MI vs. MI+DCI |  | 1.30 | 13.56 |  | -2.84 | 27.45 |  | 4.14 | 30.61 | 0.89 | 19.16 |

| **AND** |  | Direct | |  | Indirect | |  | Difference | | *P* | *tau* |
| --- | --- | --- | --- | --- | --- | --- | --- | --- | --- | --- | --- |
| Comparisons |  | Coefficient | SE |  | Coefficient | SE |  | Coefficient | SE |  |  |
| Met vs. TZDs |  | - | - |  | - | - |  | - | - | - | - |
| Met vs. MI+DCI |  | -5.00 | 43.14 |  | 3.31 | 83.06 |  | -8.31 | 93.59 | 0.93 | 43.13 |
| DCI vs. MI |  | 16.00 | 43.68 |  | -17.61 | 239.10 |  | 33.61 | 243.86 | 0.89 | 41.40 |
| MI vs. MI+DCI |  | -3.99 | 43.49 |  | -18.43 | 110.29 |  | 14.44 | 118.61 | 0.90 | 43.10 |

| **mF-G score** |  | Direct | |  | Indirect | |  | Difference | | *P* | *tau* |
| --- | --- | --- | --- | --- | --- | --- | --- | --- | --- | --- | --- |
| Comparisons |  | Coefficient | SE |  | Coefficient | SE |  | Coefficient | SE |  |  |
| Met vs. TZDs |  | - | - |  | - | - |  | - | - | - | - |
| Met vs. MI |  | 0.90 | 1.64 |  | -0.02 | 57.74 |  | 0.92 | 57.76 | 0.99 | 1.15 |
| Met vs. Met+TZDs |  | - | - |  | - | - |  | - | - | - | - |
| DCI vs. MI |  | -0.06 | 1.18 |  | 1.86 | 200.06 |  | -1.92 | 200.06 | 0.99 | 1.15 |
| MI vs. MI+DCI |  | -2.60 | 1.56 |  | -4.40 | 141.47 |  | 1.80 | 141.47 | 0.99 | 1.15 |

| **Menstrual frequency** |  | Direct | |  | Indirect | |  | Difference | | *P* | *tau* |
| --- | --- | --- | --- | --- | --- | --- | --- | --- | --- | --- | --- |
| Comparisons |  | Coefficient | SE |  | Coefficient | SE |  | Coefficient | SE |  |  |
| Met vs. TZDs |  | -0.16 | 0.44 |  | 0.41 | 3.09 |  | -0.58 | 3.12 | 0.85 | 0.86 |
| Met vs. MI |  | 0.11 | 1.14 |  | -0.58 | 1.65 |  | 0.69 | 2.01 | 0.73 | 0.83 |
| Met vs. MI+DCI |  | 2.46 | 1.16 |  | 3.15 | 1.64 |  | -0.69 | 2.01 | 0.73 | 0.83 |
| Met vs. Met+TZDs |  | 1.27 | 0.67 |  | -2.34 | 1.71 |  | 3.60 | 1.82 | 0.05 | 0.57 |
| TZDs vs. Met+TZDs |  | 0.00 | 0.96 |  | 2.05 | 1.05 |  | -2.05 | 1.43 | 0.15 | 0.68 |
| DCI vs. MI |  | -0.33 | 0.96 |  | 0.11 | 200.01 |  | -0.44 | 200.02 | 1.00 | 0.77 |
| MI vs. MI+DCI |  | 3.04 | 1.18 |  | 2.35 | 1.63 |  | 0.69 | 2.01 | 0.73 | 0.83 |

| **Gastrointestinal adverse events** |  | Direct | |  | Indirect | |  | Difference | | *P* | *tau* |
| --- | --- | --- | --- | --- | --- | --- | --- | --- | --- | --- | --- |
| Comparisons |  | Coefficient | SE |  | Coefficient | SE |  | Coefficient | SE |  |  |
| Met vs. TZDs |  | 0.53 | 0.76 |  | 0.06 | 5.06 |  | 0.47 | 5.11 | 0.93 | 1.03 |
| Met vs. MI |  | - | - |  | - | - |  | - | - | - | - |
| Met vs. BBR |  | - | - |  | - | - |  | - | - | - | - |
| Met vs. MI+DCI |  | - | - |  | - | - |  | - | - | - | - |
| Met vs. Met+TZDs |  | 0.02 | 1.30 |  | 1.20 | 4.23 |  | -1.18 | 4.42 | 0.79 | 1.01 |
| TZDs vs. Met+TZDs |  | 0.00 | 2.26 |  | -0.63 | 1.73 |  | 0.63 | 2.85 | 0.82 | 1.02 |

*^1^P* for the assessment of inconsistency. *P* <0.05 was considered to indicate a significant inconsistency existed between direct and indirect evidence.

Abbreviations: Met, Metformin; TZDs, Thiazolidinediones; MI, Myo-inositol; DCI, D-chiro-inositol; BBR, Berberine; TT, total testosterone; SHBG, sex hormone binding globulin; AND, androstenedione; mF-G score, modified Ferriman-Gallwey score; BMI, body mass index; WHR, waist-hip ratio; FPG, fasting plasma glucose; FINS, fasting insulin; HOMA-IR, Homeostatic Model Assessment of Insulin Resistance; TG, triglyceride; TC, total cholesterol; HDL-C, high density lipoprotein cholesterol; LDL-C, low density lipoprotein cholesterol;

**Appendix S2**

2.1 Forest plots in NMA

Abbreviations: Met, Metformin; TZDs, Thiazolidinediones; MI, Myo-inositol; DCI, D-chiro-inositol; BBR, Berberine; TT, total testosterone; SHBG, sex hormone binding globulin; AND, androstenedione; mF-G score, modified Ferriman-Gallwey score; BMI, body mass index; WHR, waist-hip ratio; FPG, fasting plasma glucose; FINS, fasting insulin; HOMA-IR, Homeostatic Model Assessment of Insulin Resistance; TG, triglyceride; TC, total cholesterol; HDL-C, high density lipoprotein cholesterol; LDL-C, low density lipoprotein cholesterol;

2.2 the NMA and TMA results for negative efficacy outcomes^1^

| Outcomes | Studies | Participants | Traditional pairwise meta-analysis (TMA) | | Network meta-analysis (NMA) | |
| --- | --- | --- | --- | --- | --- | --- |
|  |  |  | Heterogeneity | Effect Estimate (95% CI) | Studies | Effect Estimate (95% CI) |
| **SHBG** |  |  |  |  |  |  |
| TZDs vs Met | 3 | 149 | (*P* ＜0.00001); *I²* = 99% | 15.73 [-16.37, 47.84] | 3 | 15.89 [-3.62, 35.40] |
| MI vs Met | 1 | 60 | Not applicable | 5.20 [-6.13, 16.53] | 4 | 3.24 [-22.59, 29.08] |
| MI + DCI vs Met | 1 | 64 | Not applicable | 2.20 [0.62, 3.78] | 3 | 3.74 [-21.77, 29.24] |
| MI + DCI vs MI | 2 | 72 | (*P* = 0.19); *I²* = 43% | 1.36 [-0.01, 2.72] | 3 | 0.46 [-19.01, 19.93] |
| **AND** |  |  |  |  |  |  |
| TZDs vs Met | 4 | 172 | (*P* = 0.01); *I²* = 72% | **33.36 [0.24, 66.48]** | 4 | **41.19 [26.05, 70.38]** |
| MI vs Met | / | / | / | / | 2 | -5.78 [-19.98, 8.42] |
| MI + DCI vs Met | 1 | 64 | Not applicable | -5.00 [-6.73, -3.27] | 3 | -4.99 [-14.74, 4.91] |
| MI + DCI vs MI | 2 | 72 | (*P* = 0.23); *I²* = 29% | 1.08 [-7.67, 9.83] | 3 | 0.86 [-9.33, 11.06] |
| **mF-G score** |  |  |  |  |  |  |
| TZDs vs Met | 4 | 210 | (*P* ＜0.00001); *I²* = 92% | 0.42 [-1.43, 2.28] | 4 | 0.44 [-1.01, 1.89] |
| MI vs Met | 1 | 60 | Not applicable | 0.90 [-1.41, 3.21] | 3 | 0.10 [-3.40, 3.20] |
| MI + DCI vs Met | / | / | / | / | 2 | -2.70 [-7.29, 1.89] |
| MI + DCI vs MI | 2 | 44 | (*P* = 1.00); *I²* = 0% | -2.60 [-5.22, 0.02] | 3 | -2.60 [-5.78, 0.58] |
| **WHR** |  |  |  |  |  |  |
| TZDs vs Met | 6 | 261 | (*P* < 0.00001); *I²* = 95% | -0.00 [-0.05, 0.05] | 8 | 0.00[-0.03, 0.03] |
| TZDs + Met vs Met | 3 | 171 | (*P* = 0.15); *I²* = 48% | 0.01 [-0.02, 0.04] | 4 | 0.01 [-0.03, 0.05] |
| MI vs Met | 2 | 120 | (*P* = 1.00); *I²* = 0% | -0.01 [-0.01, 0.01] | 5 | -0.01 [-0.06, 0.05] |
| MI + DCI vs Met | / | / | / | / | 3 | 0.01 [-0.05, 0.08] |
| MI + DCI vs MI | 3 | 80 | (*P* = 0.21); *I²* = 36% | 0.02 [-0.02, 0.06] | 3 | 0.02 [-0.02, 0.07] |

^1^ *I^2^* > 50% or *P* < 0.05 indicated substantial heterogeneity.

2.3 Treatment Relative Ranking

| Treatment | SUCRA | PrBest | MeanRank |  | Treatment | SUCRA | PrBest | MeanRank |
| --- | --- | --- | --- | --- | --- | --- | --- | --- |
| **Menstrual frequency** |  |  |  |  | **Gastrointestinal adverse events** |  |  |  |
| Met | 27.8 | 0 | 4.6 |  | Met | 23.5 | 0 | 4.8 |
| TZDs | 37.7 | 0.1 | 4.1 |  | TZDs | 75.8 | 19.1 | 2.2 |
| DCI | 41.9 | 3 | 3.9 |  | MI | 72.8 | 31.6 | 2.4 |
| MI | 28.1 | 0 | 4.6 |  | BBR | 12.4 | 0.9 | 5.4 |
| MI+DCI | 98 | 91.9 | 1.1 |  | MI+DCI | 81.2 | 48.2 | 1.9 |
| TZDs+Met | 66.4 | 4.9 | 2.7 |  | TZDs+Met | 34.2 | 0.1 | 4.3 |

| Treatment | SUCRA | PrBest | MeanRank |  | Treatment | SUCRA | PrBest | MeanRank |
| --- | --- | --- | --- | --- | --- | --- | --- | --- |
| **FPG** |  |  |  |  | **FINS** |  |  |  |
| Met | 31.7 | 0 | 5.1 |  | Met | 20.7 | 0 | 5.8 |
| TZDs | 75.1 | 16.4 | 2.5 |  | TZDs | 62.4 | 8.5 | 3.3 |
| MI | 49.9 | 2.8 | 4 |  | MI | 30.7 | 1.1 | 5.2 |
| BBR | 11.9 | 1.5 | 6.3 |  | BBR | 58 | 29.5 | 3.5 |
| MI+DCI | 85.3 | 51.1 | 1.9 |  | MI+DCI | 46.3 | 8.4 | 4.2 |
| BBR+Met | 62.5 | 27.1 | 3.2 |  | BBR+Met | 68.4 | 38.9 | 2.9 |
| TZDs+Met | 33.5 | 1.2 | 5 |  | TZDs+Met | 63.5 | 13.4 | 3.2 |

| Treatment | SUCRA | PrBest | MeanRank |  | Treatment | SUCRA | PrBest | MeanRank |
| --- | --- | --- | --- | --- | --- | --- | --- | --- |
| **HOMA-IR** |  |  |  |  | **TT** |  |  |  |
| Met | 9.6 | 0 | 7.3 |  | Met | 35.3 | 0 | 5.5 |
| TZDs | 68.6 | 8.7 | 3.2 |  | TZDs | 3.9 | 0 | 7.7 |
| DCI | 45.1 | 9.2 | 4.8 |  | DCI | 26.8 | 0 | 6.1 |
| MI | 32.8 | 0 | 5.7 |  | MI | 26.9 | 0 | 6.1 |
| BBR | 52.5 | 21.7 | 4.3 |  | BBR | 79.8 | 45.8 | 2.4 |
| MI+DCI | 80.8 | 33.9 | 2.3 |  | MI+DCI | 75.4 | 3 | 2.7 |
| BBR+Met | 30.8 | 0.4 | 5.8 |  | BBR+Met | 92.2 | 51.2 | 1.5 |
| TZDs+Met | 79.9 | 26.1 | 2.4 |  | TZDs+Met | 59.7 | 0.1 | 3.8 |

| Treatment | SUCRA | PrBest | MeanRank |  | Treatment | SUCRA | PrBest | MeanRank |
| --- | --- | --- | --- | --- | --- | --- | --- | --- |
| **SHBG** |  |  |  |  | **mF-G score** |  |  |  |
| Met | 32.5 | 0.6 | 4.4 |  | Met | 46.1 | 3.7 | 3.7 |
| TZDs | 72 | 36.5 | 2.4 |  | TZDs | 30.2 | 2.4 | 4.5 |
| DCI | 45.9 | 23.4 | 3.7 |  | DCI | 44.1 | 6.7 | 3.8 |
| MI | 47.8 | 9.3 | 3.6 |  | MI | 44.9 | 1.1 | 3.8 |
| MI+DCI | 50 | 13.1 | 3.5 |  | MI+DCI | 88.6 | 73.9 | 1.6 |
| TZDs+Met | 51.8 | 17.2 | 3.4 |  | TZDs+Met | 46.1 | 12.1 | 3.7 |

| Treatment | SUCRA | PrBest | MeanRank |  | Treatment | SUCRA | PrBest | MeanRank |  | Treatment | SUCRA | PrBest | MeanRank |
| --- | --- | --- | --- | --- | --- | --- | --- | --- | --- | --- | --- | --- | --- |
| **AND** |  |  |  |  | **WHR** |  |  |  |  | **BMI** |  |  |  |
| Met | 37 | 2.8 | 3.5 |  | Met | 47 | 1.9 | 3.7 |  | Met | 52.1 | 0 | 4.4 |
| TZDs | 0 | 0 | 5 |  | TZDs | 47.7 | 7 | 3.6 |  | TZDs | 2.8 | 0 | 7.8 |
| DCI | 90.1 | 81.5 | 1.4 |  | MI | 64 | 20.3 | 2.8 |  | DCI | 62 | 15 | 3.7 |
| MI | 62.6 | 7.8 | 2.5 |  | MI+DCI | 29.3 | 5.8 | 4.5 |  | MI | 30.4 | 0 | 5.9 |
| MI+DCI | 60.3 | 7.9 | 2.6 |  | BBR+Met | 79.6 | 60.5 | 2 |  | BBR | 65.7 | 17.8 | 3.4 |
|  |  |  |  |  | TZDs+Met | 32.3 | 4.6 | 4.4 |  | MI+DCI | 54.5 | 1.5 | 4.2 |
|  |  |  |  |  |  |  |  |  |  | BBR+Met | 94.2 | 65.6 | 1.4 |
|  |  |  |  |  |  |  |  |  |  | TZDs+Met | 38.3 | 0 | 5.3 |

| Treatment | SUCRA | PrBest | MeanRank |  | Treatment | SUCRA | PrBest | MeanRank |
| --- | --- | --- | --- | --- | --- | --- | --- | --- |
| **TG** |  |  |  |  | **TC** |  |  |  |
| Met | 37.9 | 0 | 4.1 |  | Met | 38.2 | 1 | 4.1 |
| TZDs | 12.3 | 0 | 5.4 |  | TZDs | 52.1 | 7.7 | 3.4 |
| MI | 58.8 | 0.3 | 3.1 |  | MI | 40.2 | 8.7 | 4 |
| BBR | 17.6 | 2.2 | 5.1 |  | BBR | 62.4 | 39.7 | 2.9 |
| MI+DCI | 76.6 | 5.8 | 2.2 |  | MI+DCI | 50.3 | 23.1 | 3.5 |
| TZDs+Met | 96.7 | 91.7 | 1.2 |  | TZDs+Met | 56.9 | 19.8 | 3.2 |

| Treatment | SUCRA | PrBest | MeanRank |  | Treatment | SUCRA | PrBest | MeanRank |
| --- | --- | --- | --- | --- | --- | --- | --- | --- |
| **HDL** |  |  |  |  | **LDL** |  |  |  |
| Met | 41.8 | 0 | 3.9 |  | Met | 25.8 | 0 | 4.7 |
| TZDs | 89.9 | 61.5 | 1.5 |  | TZDs | 88.3 | 50.4 | 1.6 |
| MI | 50.6 | 8.2 | 3.5 |  | MI | 16.6 | 0 | 5.2 |
| BBR | 11.6 | 2.5 | 5.4 |  | BBR | 59 | 39 | 3 |
| MI+DCI | 46.6 | 16.6 | 3.7 |  | MI+DCI | 53.1 | 3.7 | 3.3 |
| TZDs+Met | 59.6 | 11.1 | 3 |  | TZDs+Met | 57.2 | 6.9 | 3.1 |

Abbreviations: Met, Metformin; TZDs, Thiazolidinediones; MI, Myo-inositol; DCI, D-chiro-inositol; BBR, Berberine; TT, total testosterone; SHBG, sex hormone binding globulin; AND, androstenedione; mF-G score, modified Ferriman-Gallwey score; BMI, body mass index; WHR, waist-hip ratio; FPG, fasting plasma glucose; FINS, fasting insulin; HOMA-IR, Homeostatic Model Assessment of Insulin Resistance; TG, triglyceride; TC, total cholesterol; HDL-C, high density lipoprotein cholesterol; LDL-C, low density lipoprotein cholesterol;
